# A Secure Spatial Multiplexing Transmission Scheme in MIMO Amplify-and-Forward Wiretap Relaying Systems Using Deliberate Precoder Randomization

**DOI:** 10.3390/s26030860

**Published:** 2026-01-28

**Authors:** Kyunbyoung Ko, Changick Song

**Affiliations:** Department of Electronic Engineering, Korea National University of Transportation, 50 Daehak-ro, Chungju-si 27469, Republic of Korea; kbko@ut.ac.kr

**Keywords:** physical layer security, AFF, MIMO AF relay, spatial multiplexing, MMSE

## Abstract

Physical-layer security offers low probability of interception (LPI) in wireless communication systems. While prior methods such as the directional beamforming and secrecy coding schemes require knowledge of the eavesdropper (Eve)’s channel, passive eavesdropping limits their practicality. Artificial additive noise and artificial fast fading (AFF) schemes address the issue by degrading detection ability of a potential Eve without knowing its channel information. In particular, AFF achieves LPI by effectively shortening the coherence time of Eve’s channel using a random precoder while keeping the legitimate receiver (Bob)’s channel deterministic. In this paper, we propose a novel AFF design for spatial multiplexing multi-input multi-output (MIMO) amplify-and-forward (AF) relay systems. First, we formulate an optimization problem to achieve minimum mean squared error (MMSE) of Bob’s signals while guaranteeing LPI conditions from Eve, which is generally non-convex. To tackle the non-convexity of the problem, we apply a convex set approximation technique and thereby derive a simple closed-form design. Finally, we evaluated the performance of both Bob and Eve via computer simulations to demonstrate the effectiveness of our proposed design.

## 1. Introduction

With the rapid proliferation of wireless radio technologies, security in wireless communications has become a critical challenge in both personal and professional domains. Unlike wired communication systems, wireless links inherently lack physical boundaries, allowing any nearby receiver to potentially intercept transmitted signals. Physical-layer security aims to safeguard the radio interface by achieving low probability of interception (LPI) while remaining independent of, yet fully compatible with, upper-layer encryption techniques.

Since the pioneering work of Wyner [1], the concept of a secrecy rate has widely been utilized to measure secrecy performance and derive a rate bound for secure transmission in the physical layer. Although designs and analyses based on the secrecy rate provide useful insight into the secrecy behavior of a communication system, they entail several practical limitations. Primarily, the secrecy rate is required for the transmitter (Alice) to have perfect or statistical knowledge of the eavesdropper’s (Eve’s) channel state information (CSI). However, this is generally unrealizable in practice since passive Eve cannot cooperate with Alice to perform the channel feedback. In addition, a positive secrecy rate is only achievable with relative signal-to-noise ratio (SNR) advantage at the legitimate receiver (Bob) compared to Eve, which is difficult to guarantee in practice. Moreover, the transmit signal is usually required to follow a Gaussian distribution, which hardly captures the effect of practical modulation and coding schemes. It has been shown that the information leakage to Eve can be driven to zero without the aid of Gaussian codewords, either by transmitting the signals in the orthogonal direction of Eve’s channel via multi-antenna beamforming [2] or by employing random binning-based secrecy coding schemes [3,4]. However, such schemes still demand Eve’s CSI at Alice.

To address this issue, artificial noise (AN) transmission schemes have been actively investigated in recent years [5,6]. Unlike the aforementioned approaches, the primary objective of AN is to degrade the detection capability of potential Eves with the absence of the Alice-to-Eve link CSI, while minimizing its impact on Bob’s reception, rather than to completely eliminate the information leakage to Eve. One way to implement such a scheme is to transmit AN in the orthogonal direction of Bob’s channel using extra degrees of freedom in the transmit antenna dimension and thereby degrade Eve’s received SNR while allowing Bob to effectively extract information.

Since orthogonal AN was first theoretically established by Goel and Negi [7], a variety of AN methods have been developed in the literature. For example, the authors in [8,9] proposed non-orthogonal AN techniques that can further interfere with Eve by sacrificing Bob’s communication quality, thereby increasing the secrecy rate. However, such a scheme requires Alice to know the CSI of Eve. In [10,11], the authors proposed the constructive AN schemes that can be designed at a symbol-rate to be constructive to Bob while being destructive to Eve. The AN-aided on–off transmission scheme was developed in [12] considering the limited training and feedback overhead. In [13], the authors addressed data carrying AN, in which encrypted message symbols with pre-shared secret keys act as interference to the unintended users like Eve. From Eve’s point of view, the artificial noise elimination (ANE) schemes have also been actively investigated to counteract AN with and without knowing the Alice-to-Bob link CSI [14,15,16,17]. The previous studies have shown that Eve with a sufficiently large antenna array and adequate channel knowledge can significantly suppress the impact of AN.

In the meantime, there has been growing interest in the artificial fast fading (AFF) designs as a countermeasure against Eve’s advanced ANE techniques. In the AFF strategy, Alice basically transmits no downlink training to prevent Eve from estimating its own channel and noise distribution, which are essential knowledge for coherent detection, whereas Alice obtains the Alice-to-Bob link CSI through the uplink training from Bob. In this situation, a primary goal of AFF is to effectively shorten the coherence time of Eve’s channels in order to invalidate Eve’s non-coherent blind estimation schemes. To this end, one can design a random precoder which deliberately enhances fluctuation of Eve’s channel while making Bob’s channel deterministic over a channel coherence duration by exploiting the excess degrees of freedom (DoFs) at Alice. Since AFF was first developed in multiple-input single-output (MISO) wiretap channels [18], it has been extended to multi-carrier [19], MIMO [20], and multi-user MISO environments [21]. In addition, the authors in [22] analyzed the performance of AFF and compared the results with AN in terms of the secrecy rate. It has been further demonstrated in [23] that even if Eve has full knowledge of CSI of all nodes, information leakage to Eve can be effectively prevented as long as the AFF precoder remains fully random.

Cooperative relaying has also been considered as a promising technique to improve the security performance in the physical layer [24]. To further enhance security and practical feasibility, cooperative jamming techniques based on AN have widely been investigated in the literature. For example, the authors in [25] studied orthogonal AN in secure MIMO amplify-and-forward (AF) relaying systems, where both Alice (or the source) and the relay broadcasts multi-dimensional AN while simultaneously transmitting a single data stream to Bob (or the destination). In [26,27], it was shown that we can further interfere with Eve by allowing the destination to transmit jamming signals concurrently with the message transmission from Alice. In [28], various AN-aided transmission schemes were developed in a two-way MIMO AF relaying system with different security–complexity trade-offs. Cooperative jamming is also useful for impairing potential eavesdropping at untrusted relays [29,30]. In [31,32], the authors exploited cooperative jamming to proactively wiretap the communication between two suspicious users.

In wireless relaying systems, downlink training is inevitable even in time division duplexing operation. This is because no downlink training from Alice would require pre-equalization of the Alice-to-relay channel, which becomes highly inefficient when the number of relay antennas exceeds that of Alice. Also, the uplink training from the relay immediately serves as the downlink training to both Bob and Eve due to the nature of wireless transmission. For these reasons, most existing studies on relay-assisted physical-layer security typically presume the use of downlink training at both Alice and the relay and further assume that Bob and Eve are aware of all CSI associated with themselves. As discussed before, such channel knowledge provides Eve with useful information for performing ANE to mitigate the effect of jamming. Nevertheless, studies addressing ANE issues in the secure MIMO relay systems remain scarce in the literature.

In this paper, we highlight the vulnerability of AN-based jamming to ANE in the relaying systems and propose a novel AFF design as an effective alternative. Specifically, we investigate AFF-based relay transceiver designs for secure spatial multiplexing MIMO AF relaying systems, where Alice transmits multiple data streams simultaneously to Bob with the aid of a trusted multi-antenna relay at the presence of a potential Eve at each hop. The proposed design aims to satisfy two conditions simultaneously, which we refer to as the *LPI conditions*. One condition is to maintain Bob’s channel constant over a channel coherence block length, while the remaining condition is to enhance the security by incurring unresolvable randomness in Eve’s channels. To achieve these two conditions simultaneously, first we apply the random AFF precoding scheme [23] at Alice to secure the first-hop channel. Then, we design the relay transceiver such that the randomness of the first-hop channel is propagated to the relay-to-Eve channel while having no impact on Bob’s detection behavior.

To further enhance the performance of Bob, we shape Bob’s effective channel such that the total mean squared error (MSE) between the signals and its estimates is minimized under the LPI constraints. Such a minimum MSE (MMSE) problem is generally non-convex and thus is difficult to solve. To solve the problem, we first propose a gradient descent (GD)-based iterative optimization method that can provide near optimal performance. To address the high computational complexity of such an iterative algorithm and provide useful insights into the system, we also identify a low-complexity closed-form solution based on a convex-hull relaxation technique. It is worth noting that both AFF designs provide security without requiring disruptive changes to the training structure of the conventional unsecured MMSE relaying systems [33,34].

In contrast to conventional secrecy-rate-based frameworks, we adopt a signal processing oriented approach that focuses on the error performance of Bob and Eve. This approach enables the evaluation of the security gain even in the presence of a powerful eavesdropper equipped with a large number of antennas and capable of performing ANE, with which a positive secrecy rate is generally not guaranteed. Note that such an approach provides insights from a practical implementation perspective and is particularly useful in hardware-constrained environments, such as sensor networks and Internet of Things (IoT) networks, where assessing the reliability of data reception at Bob and Eve is often more critical than characterizing the theoretical limits on transmission rates [35,36]. In light of this, extensive simulation results are provided in terms of bit error rate (BER) performance of Bob and Eve to demonstrate the effectiveness of the proposed design. Our simulation results effectively capture the influence of pragmatic system parameters, including modulation schemes, detection strategies, and the amount of CSI at the receivers. We also confirm from the results that while conventional AN-based jamming techniques are highly vulnerable to ANE attacks, the proposed AFF scheme is inherently robust against such attacks.

**Organization**: The remainder of the paper is organized as follows. In Section 2, we describe the wiretap relay channel model considered in this paper. In Section 3, we provide preliminary remarks, and in Section 4, we describe the design outline and propose a closed-form transceiver design. Section 5 provides the simulation results, and Section 6 concludes the paper.

**Notations**: Throughout the paper, normal letters represent scalar quantities, boldface letters indicate vectors, and boldface uppercase letters designate matrices. The superscripts ${\left(\cdot \right)}^{T}$, ${\left(\cdot \right)}^{*}$, and ${\left(\cdot \right)}^{H}$ stand for the transpose, conjugate, and conjugate transpose operations. We define ⊗ as a Kronecker product, and E[·] and $E_{x}\left[\cdot \right]$ denote the expectation operators taken over all random variables and a specific random variable *x*, respectively, while R and C designate sets of real and complex numbers, respectively. Also, we define ${∥A∥}_{F}$ and $\mathrm{Tr}\left(A\right)$ as the Frobenius norm and trace of a matrix A, respectively, and $\mathrm{blkdiag}[A_{1},\ldots ,A_{K}]$ stands for the block-wise diagonal matrix with sub-matrices ${\left\{A_{k}\right\}}_{k=1}^{K}$ on its main diagonal. The N×N identity and zero matrices are denoted by $I_{N}$ and $0_{N}$, respectively. A notation x∼CN(p,q) represents that all elements of x are the independent and identically distributed (i.i.d.) circularly symmetric complex Gaussian with mean *p* and variance *q*. Finally, we denote x(n) the *n*-th realization of a random variable *x*.

## 2. System Model

**Figure 1:**
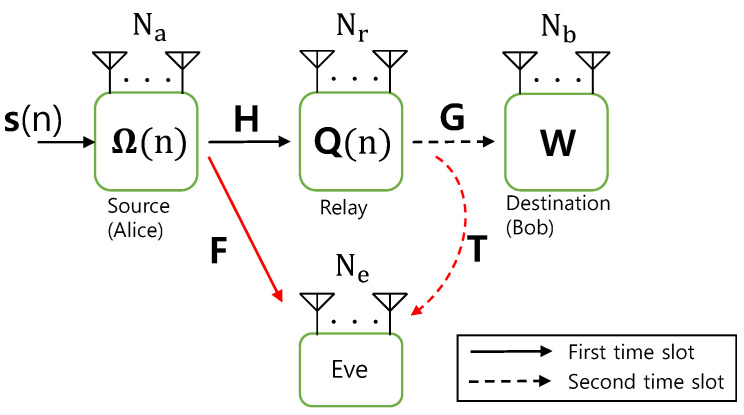
A schematic diagram for MIMO AF relaying systems with wiretap channels and deliberate precoder randomization (Red arrows indicate wiretap channels to Eve).

As described in Figure 1, we consider a cooperative relaying system, where a multi-antenna AF relay helps secure communications between the source (Alice) and the destination (Bob) given the existence of a passive eavesdropper. We assume that the direct path between Alice and Bob can be ignored due to large path loss (Extension of our work to the scenarios of non-negligible direct link remains open for future works). However, all the links to Eve cannot be ignored as we have no information on Eve’s location. Alice, the relay, Bob, and Eve are equipped with $N_{a}$, $N_{r}$, $N_{b}$, and $N_{e}$ antennas, which implies that in the base-band representation and the Alice-to-relay, Alice-to-Eve, relay-to-Bob, and relay-to-Eve channels are denoted by complex matrices $H\in C^{N_{r}\times N_{a}}$, $F\in C^{N_{e}\times N_{a}}$, $G\in C^{N_{b}\times N_{r}}$, and $T\in C^{N_{e}\times N_{r}}$, respectively.

We consider a block-wise fading scenario, where all channel coefficients remain invariant over a coherent fading block-length *N*, after which they may change to new random values. Considering an inter-element spacing of at least half a wavelength among all antennas, all channel matrices and their elements are assumed to be independent of each other. In this paper, we adopt the spatial multiplexing transmission scheme, where Alice transmits $N_{s}$ data streams at the same time with $N_{s}\leq \min\left(N_{a},N_{r},N_{b},N\right)$. However, to avoid the loop interference at the relay, each data transmission at Alice and the relay occurs in two separate time slots.

In this paper, we assume that one time slot equals channel coherence block length *N*, but the results can be applied to more general scenarios. In the first time slot, Alice generates an input sequence S=[s(1),…,s(N)], where $s\left(n\right)\in C^{N_{s}}$ represents a message symbol vector in the *n*-th symbol interval, which meets E[s]=0 and $\mathrm{Tr}\left(R_{s}\right)=N_{s}$ for transmit signal covariance $R_{s}≜E\left[ss^{H}\right]$ and then transmits a precoded signal $x_{A}\left(n\right)=P\Omega \left(n\right)s\left(n\right)\in C^{N_{s}}$ which is constrained by its maximum power budget as $E[∥x_{A}{∥}^{2}]\leq P_{A}$. Here, $P\in C^{N_{a}\times N_{s}}$ denotes a precoding matrix that is customized to the Alice-to-relay channel H, whereas $\Omega \left(n\right)\in C^{N_{s}\times N_{s}}$ indicates a random AFF precoder, which is designed to artificially shorten coherence time of Eve’s effective channels. We assume that each coefficient of Ω varies in every T≥1 symbol periods under the condition that $E\left[\Omega \Omega^{H}\right]=I_{N_{s}}$. Then, the received signals at the relay and Eve are expressed by

(1) $$\begin{array}{ccc} y_{R}\left(n\right) & = & Hx_{A}\left(n\right)+n_{R}\left(n\right) \end{array}$$

(2) $$\begin{array}{ccc} \mathrm{and}\;\;y_{E_{1}}\left(n\right) & = & Fx_{A}\left(n\right)+n_{E_{1}}\left(n\right), \end{array}$$

where $n_{R}\left(n\right)\in C^{N_{r}}$ and $n_{E_{1}}\left(n\right)\in C^{N_{e}}$ designate the noise vectors at the relay and Eve in the *n*-th symbol interval, respectively.

In the next time slot, the relay received signal $y_{R}\left(n\right)$ in (1) is linearly amplified by a relay transceiver $Q\left(n\right)\in C^{N_{r}\times N_{r}}$ and then forwarded to Bob with a power constraint $E[∥x_{R}{∥}^{2}]\leq P_{R}$ where $x_{R}\left(n\right)=Q\left(n\right)y_{R}\left(n\right)$ signifies the relay transmit signal. Then, the received signals at Bob and Eve in the *n*-th symbol interval are, respectively, written as

(3) $$\begin{array}{ccc} y_{B}\left(n\right) & = & Gx_{R}\left(n\right)+n_{B}\left(n\right) \end{array}$$

(4) $$\begin{array}{ccc} \mathrm{and}\;\;y_{E_{2}}\left(n\right) & = & Tx_{R}\left(n\right)+n_{E_{2}}\left(n\right), \end{array}$$

where $n_{B}\left(n\right)\in C^{N_{b}}$ and $n_{E_{2}}\left(n\right)\in C^{N_{e}}$ designate the noise vectors at Bob and Eve in the second time slot, respectively. Without loss of generality, we assume that all elements of the noise vectors $n_{R}$, $n_{B}$, $n_{E_{1}}$, and $n_{E_{2}}$ are independent and identically distributed complex Gaussian, i.e., $\mathrm{CN}(0,\sigma_{n}^{2})$, throughout the paper. Finally, the estimated signal waveform at Bob is expressed as $z\left(n\right)=Wy_{B}\left(n\right)\in C^{N_{s}}$ with a linear receiver $W\in C^{N_{s}\times N_{b}}$. In the remainder of this paper, we omit the symbol index *n* whenever the corresponding signal is treated as a random variable.

All legitimate nodes are allowed to transmit training (or pilot) signals for channel estimation, as in conventional systems. Accordingly, the relay acquires full CSI of both H and G through uplink and downlink training with Alice and Bob. In contrast, Bob and Eve can access only the CSI of their respective links, i.e., G and {F,T}, since the relay does not forward any information about H to subsequent nodes. Similarly, Alice is assumed to know only the first-hop channel H. As the passive eavesdropper may not transmit any signals, it is reasonable to assume that Eve’s channels F and T are unknown to all legitimate nodes. The amount of CSI at each node is summarized in Table 1. In addition, a potential eavesdropper, whose location is unknown to the legitimate nodes, is typically away from the legitimate nodes. Thus, we assume that Eve’s eavesdropping channels F and T are statistically uncorrelated with the legitimate link channels H and G, respectively.

In this paper, we consider a trusted and dedicated relay system, in which a secret key-based cryptographic system can facilitate secure communication between Alice and Bob. In contrast, for Bob, which represents a relatively lightweight device such as an IoT node, we assume that secret key sharing is not feasible due to the high overhead required for the key distribution process. In this circumstance, it is difficult for Alice to directly encrypt the messages using a secret key, but such a key can still be exploited to enhance physical-layer security. In this paper, we assume that a shared secret key between Alice and the relay enables each node to locally and securely generate an identical random precoder sequence ${\left\{\Omega \left(n\right)\right\}}_{n=1}^{N}$ in advance.

To implement this process, the public-key methods such as the elliptic curve Diffie-Hellman algorithm can provide an assistance to share secret information such as the session key between Alice and the relay as conducted in the current transport layer security protocols. Although the AF relay usually operates at the physical layer for signal forwarding, higher-layer protocols can be implemented for control and management purposes. We can also consider the secret-key agreement methods from wireless measurements between Alice and the relay, which are mainly performed in the physical layer [13]. Once a secret key has been agreed upon, Alice and the relay can locally produce the same sequence of AFF precoders through a cryptographically secure pseudo-random number generator [37] by exploiting the shared secret key as a seed number.

## 3. Preliminaries

This section provides a brief overview of the secure and non-secure transceiver designs that are most relevant to the proposed approach in spatial multiplexing MIMO AF relaying systems. As we consider non-AFF scenarios in this section, we temporarily assume that $\Omega \left(n\right)=I_{N_{s}}$.

### 3.1. Unsecured MMSE Designs

In non-secure transmission scenarios, joint optimization of the source, the relay, and the destination in terms of the MMSE criterion has been extensively studied in the literature [38,39]. However, a common drawback of such optimal designs is that they require CSI of all hops at both the source and the destination. To this end, the relay must forward the channel information of the previous hop to the next node in addition to transmitting its own pilot signals, which leads to increased system overhead. A solution to such a problem was later addressed in [33,34], whose main idea is succinctly summarized in the following lemma.

**Lemma 1.**  *From the MMSE point of view, the optimal relay transceiver employs a structure of Q=BL, where $L={\left(P^{H}H^{H}HP+\sigma_{n}^{2}R_{s}^{-1}\right)}^{-1}P^{H}H^{H}$ represents the Wiener filter receiver that equalizes the first hop effective channel HP and $B\in C^{N_{r}\times N_{s}}$ serves as a relay precoder. Then, the MMSE problem between s and z can be decomposed into two separate problems as*

(5) $$\begin{array}{cc} \min_{P,Q,W} & {E[∥z-s∥}^{2}]\overset{\left(a\right)}{=}\min_{P,B,W}\{E\left[{∥y-s∥}^{2}\right]+E\left[{∥z-y∥}^{2}\right]\}\\ \; & \overset{\left(b\right)}{=}\min_{P,B}\left\{Tr[{\left(\sigma_{n}^{-2}P^{H}H^{H}HP+R_{s}^{-1}\right)}^{-1}]+Tr\left[{\left(\sigma_{n}^{-2}B^{H}G^{H}GB+R_{y}^{-1}\right)}^{-1}\right]\right\}\\ \; & ≃\min_{P}Tr[{\left(\sigma_{n}^{-2}P^{H}H^{H}HP+R_{s}^{-1}\right)}^{-1}]+\min_{B}Tr[{\left(\sigma_{n}^{-2}B^{H}G^{H}GB+R_{s}^{-1}\right)}^{-1}], \end{array}$$

*where $y\left(n\right)≜Ly_{R}\left(n\right)$ and $R_{y}≜E\left[yy^{H}\right]$. The equality (a) follows from the orthogonality principle, and (b) is obtained by applying the optimal destination receiver*

(6) $$\begin{array}{cc} W & ={\left(B^{H}G^{H}GB+\sigma_{n}^{2}R_{y}^{-1}\right)}^{-1}B^{H}G^{H},\\ \; & ≃{\left(B^{H}G^{H}GB+\sigma_{n}^{2}R_{s}^{-1}\right)}^{-1}B^{H}G^{H} \end{array}$$

*which is approximated as in (6) due to the fact that $R_{y}≃R_{s}$ in the high SNR regime. As a result, we have (5).*

It has been shown that the approximation $R_{y}≃R_{s}$ is fairly accurate even in the low-to medium SNR range, which implies that the destination can detect the signals exploring only the second-hop CSI G. Also, the CSI of the first hop channel is sufficient for the source precoder design. In this case, the relay only needs to transmit its own pilots without having to forward the previous link CSI to the next hop, and thus we can substantially reduce the overall training overhead. Finally, the source and relay precoders P and B can be separately optimized with respective power constraints $E[∥x_{A}{∥}^{2}]\leq P_{A}$ and $E[∥x_{R}{∥}^{2}]\leq P_{R}$ as detailed in prior studies [33].

### 3.2. AN-Based Secure MMSE Designs

AN-based secure relaying systems have been investigated in [25] assuming a single stream transmission. Although it is not difficult to generalize this to the spatial multiplexing scenarios, such a generalization has not yet been explicitly discussed from the MMSE point of view in the literature. In the following, we briefly describe how AN can be integrated to the MMSE-based MIMO AF relaying systems and how it can be vulnerable to ANE attack from Eve.

Considering orthogonal AN that does not require Eve’s CSI at the transmitter sides, we can simply inject the AN jamming vectors $d_{a}\in C^{N_{a}-N_{s}}$ and $d_{r}\in C^{N_{r}-N_{s}}$ into the source and relay transmit signals as

$$\begin{array}{cc} x_{A}\left(n\right) & =\sqrt{\nu_{a}}Ps\left(n\right)+\sqrt{1-\nu_{a}}P_{n}d_{a}\left(n\right)\\ x_{R}\left(n\right) & =\sqrt{\nu_{r}}By\left(n\right)+\sqrt{1-\nu_{r}}B_{n}d_{r}\left(n\right), \end{array}$$

where $0\leq \nu_{a}\leq 1$ and $0\leq \nu_{r}\leq 1$ imply the power allocation factors between the information bearing signals and artificial jamming noise at Alice and the relay, respectively. Note that we consider i.i.d. Gaussian jamming, i.e., $d_{a}∼\mathrm{CN}\left(0,P_{A}\right)$ and $d_{r}∼\mathrm{CN}\left(0,P_{R}\right)$, as it is known as the worst possible interference in terms of Eve. Here, $P_{n}\in C^{N_{a}\times \left(N_{a}-N_{s}\right)}$ and $B_{n}\in C^{N_{r}\times \left(N_{r}-N_{s}\right)}$ denote the orthonormal beamforming matrices that steer the jamming signals into the null-space of the effective channel matrices $LH\in C^{N_{s}\times N_{a}}$ and $WG\in C^{N_{s}\times N_{r}}$, respectively, such that they do not appear in the MMSE estimate process at both the relay and Bob. Therefore, the remaining procedure of the transceiver designs of P, Q, and W simply follow the approach in Lemma 1 under the modified power constraints $E[∥x_{A}{∥}^{2}]\leq \nu_{a}P_{A}$ and $E[∥x_{R}{∥}^{2}]\leq \nu_{r}P_{R}$ without considering AN. Note that this scheme includes the unsecured MMSE design in Section 3.1 as a special case with ν=0.

In contrast, the received signals at Eve are adversely affected by the jamming signals in both time slots as

$$\begin{array}{cc} y_{E,1}\left(n\right) & =Fx_{A}\left(n\right)+n_{E_{1}}\left(n\right)\\ \; & =\sqrt{\nu_{a}}FPs\left(n\right)+\sqrt{1-\nu_{a}}FP_{n}d_{a}\left(n\right)+n_{E_{1}}\left(n\right)\\ y_{E,2}\left(n\right) & =Tx_{R}\left(n\right)+n_{E_{2}}\left(n\right)\\ \; & =\sqrt{\nu_{r}}TBy\left(n\right)+\sqrt{1-\nu_{r}}TB_{n}d_{r}\left(n\right)+n_{E_{2}}\left(n\right),\\ \; & =\sqrt{\nu_{r}}TQHPs\left(n\right)+\sqrt{\nu_{r}}TQn_{R}\left(n\right)+\sqrt{1-\nu_{r}}TB_{n}d_{r}\left(n\right)+n_{E_{2}}\left(n\right). \end{array}$$

Eve may combine these two vectors into a single stacked vector to enhance its detection capability, given by

(7) $$\begin{array}{c} y_{E}\left(n\right)≜[\begin{array}{c} y_{E,1}\\ y_{E,2} \end{array}]=T_{\mathrm{an}}s\left(n\right)+n_{\mathrm{an}}\left(n\right), \end{array}$$

where

$$\begin{array}{c} T_{\mathrm{an}}≜[\begin{array}{c} \sqrt{\nu_{a}}FP\\ \sqrt{\nu_{r}}TQHP \end{array}]\;\;\mathrm{and}\;\;n_{\mathrm{an}}\left(n\right)≜T_{n}d\left(n\right)+[\begin{array}{c} n_{E_{1}}\left(n\right)\\ \sqrt{\nu_{r}}TQn_{R}\left(n\right)+n_{E_{2}}\left(n\right) \end{array}]\in C^{2N_{e}} \end{array}$$

denote Eve’s effective channel and noise, respectively, with a stacked AN vector $d\left(n\right)={\left[\begin{array}{c} d_{a}^{T}\left(n\right)\;d_{r}^{T}\left(n\right) \end{array}\right]}^{T}\in C^{M}$ and the corresponding channel

$$\begin{array}{cc} T_{n} & =[\begin{array}{cc} \sqrt{1-\nu_{a}}FP_{n} & 0\\ 0 & \sqrt{1-\nu_{r}}TB_{n} \end{array}]\in C^{2N_{e}\times M}, \end{array}$$

where $M=N_{a}+N_{r}-2N_{s}$ signifies the effective AN dimension.

It follows from (7) that compared to Bob, Eve’s SNR may deteriorate due to the jamming noise $T_{w}d\left(n\right)$. Under certain conditions, however, Eve can substantially mitigate the effect of the jamming noise, thereby increasing the probability of message interception. Further details are discussed in the subsequent subsection.

### 3.3. ANE Attack from Eve

In order to counteract the influence of AN, several ANE techniques have been proposed in terms of Eve’s perspective [14,15,16,17]. In this subsection, we employ the minor component analysis (MCA) technique, which is known as a powerful ANE method in the situation where Eve can estimate its effective noise covariance (ENC), i.e., $R_{\mathrm{an}}≜E\left[n_{\mathrm{an}}n_{\mathrm{an}}^{H}\right]$, and is equipped with a sufficiently large number of antennas such that $N_{e}\geq \left\lceil \frac{1}{2}\left(M_{E}+N_{s}\right)\right\rceil$. Here, $M_{E}$ denotes Eve’s choice for the AN dimension, which may or may not differ from *M*.

Specifically, let us define the ED of $R_{\mathrm{an}}$ as

(8) $$\begin{array}{c} R_{\mathrm{an}}=[\begin{array}{cc} U_{\mathrm{ma}} & U_{\mathrm{mi}} \end{array}][\begin{array}{cc} \Lambda_{\mathrm{ma}} & 0\\ 0 & \Lambda_{\mathrm{mi}} \end{array}][\begin{array}{c} U_{\mathrm{ma}}^{H}\\ U_{\mathrm{mi}}^{H} \end{array}], \end{array}$$

where $U_{\mathrm{ma}}\in C^{2N_{e}\times M_{E}}$ and $U_{\mathrm{mi}}\in C^{2N_{e}\times \left(2N_{e}-M_{E}\right)}$ denote the unitary eigenvector matrices corresponding to the $M_{E}$ largest and $2N_{e}-M_{E}$ smallest eigenvalues arranged in a descending order in the diagonal eigenvalue matrices $\Lambda_{\mathrm{ma}}\in C^{M_{E}\times M_{E}}$ and $\Lambda_{\mathrm{mi}}\in C^{\left(2N_{e}-M_{E}\right)\times \left(2N_{e}-M_{E}\right)}$, respectively.

Then, the MCA attempts to eliminate AN by projecting the observed vectors $y_{E}\left(n\right)$ in (7) to the direction of the $2N_{e}-M_{E}$ smallest eigenvalues of ENC by left-multiplying a projection matrix $W_{\mathrm{mca}}=\Lambda_{\mathrm{mi}}^{-1/2}U_{\mathrm{mi}}^{H}$ to $y_{E}\left(n\right)$ as

(9) $$\begin{array}{c} y_{E}^{\prime }\left(n\right)=W_{\mathrm{mca}}y_{E}\left(n\right)={\bar{T}}_{\mathrm{an},\mathrm{eff}}s\left(n\right)+n_{w}\left(n\right), \end{array}$$

where ${\bar{T}}_{\mathrm{an}}≜W_{\mathrm{mca}}T_{\mathrm{an}}$ and $n_{w}\left(n\right)≜W_{\mathrm{mca}}n_{\mathrm{an}}\left(n\right)$ stand for the projected effective channel and noise, respectively. It can be observed that $U_{\mathrm{mi}}^{H}$ provides an orthonormal projection onto the subspace corresponding to the $2N_{e}-M_{E}$ smallest eigenvalues, and $\Lambda_{\mathrm{mi}}^{-1/2}$ renders the projected effective noise white Gaussian, i.e., $n_{w}∼\mathrm{CN}\left(0,1\right)$. Such a noise whitening operation further simplifies the estimation process at Eve.

It is noteworthy that the noise covariance may be estimated by Eve using noise-only samples collected during signal-free intervals or from the residual signals after subtracting the estimated signal components. However, both approaches are challenging to implement in practice in the aforementioned training and jamming strategies since Eve can access only the wiretap channels F and T, which is not sufficient for coherent signal estimation, and further noise-only intervals are unlikely to occur. Nevertheless, from a conservative perspective, it is still crucial to investigate the detection capability of such strong Eve to account for the worst-case scenario. Although Eve still lacks complete knowledge of the effective channel ${\bar{T}}_{\mathrm{an}}$, the channel stability over a coherence time enables signal detection via blind estimation techniques.

## 4. Proposed AFF-Based Secure MMSE Designs

In this section, we propose an AFF-based secure design for MMSE-based MIMO AF relaying systems as an effective alternative to AN. One of our design goals is to achieve two LPI conditions simultaneously in the two-hop relaying channels, i.e., to generate random fluctuation on both of Eve’s channels while maintaining Bob’s effective channel constant over a coherence block length. Then, we further aim to design transmit and receive filters in each legitimate node in terms of the MMSE criterion under the LPI conditions.

As we adopt the AFF precoder Ω at Alice, which remains fully random in the first hop link, the remaining problem is to achieve the LPI in the second hop link via well-designed relay transceivers. According to the LPI condition, the randomness of the first-hop channel should propagate to Eve’s channel in the second hop while having no impact on Bob’s channel, which implies that Bob must be able to perform coherent detection using only the second-hop CSI. To address such an issue, we have to generalize the idea in Section 3.1 to a secured version in terms of AFF.

Before we proceed further, let us evolve some notations in more detail. First, we define the effective channel in the *n*-th symbol interval of the first hop link as $\bar{H}\left(n\right)≜HP\Omega \left(n\right)$. Then, we can evolve the notations as $\bar{H}\left(n\right)={\left[{\bar{H}}_{1}^{T}\left(n\right),\ldots ,{\bar{H}}_{K}^{T}\left(n\right)\right]}^{T}$ and $y_{R}\left(n\right)={\left[y_{R_{1}}^{T}\left(n\right),\ldots ,y_{R_{K}}^{T}\left(n\right)\right]}^{T}$, where ${\bar{H}}_{k}\left(n\right)\in C^{\frac{N_{r}}{K}\times N_{s}}$ and $y_{R_{k}}\left(n\right)\in C^{\frac{N_{r}}{K}}$ denote the *k*-th sub-matrix(or -vector) of $\bar{H}\left(n\right)$ and $y_{R}\left(n\right)$, respectively. Note that the number of sub-matrices K≥1 serves as a system parameter for adjusting the security level, which is chosen among the factors of $N_{r}$ in the range of $1\leq K\leq \frac{N_{r}}{N_{b}}$. As will be shown later, the security level improves as *K* increases, whereas the system reduces to the non-secure design [33] when K=1. Thus, the proposed work generalizes the previous unsecured design to a secured version. Similarly, we define the *k*-th sub-matrices of $G=[G_{1},\ldots ,G_{K}]$ and $T=[T_{1},\ldots ,T_{K}]$ as $G_{k}\in C^{N_{b}\times \frac{N_{r}}{K}}$ and $T_{k}\in C^{N_{e}\times \frac{N_{r}}{K}}$, respectively.

### 4.1. Problem Formulation

To achieve the LPI conditions for both Bob and Eve, the relay transceiver must be configured to vary in accordance with the update period of the AFF precoder. Also, we need to set the relay transceiver to be a block-diagonal form as

(10) $$\begin{array}{c} Q\left(n\right)=B_{\mathrm{bd}}L_{\mathrm{bd}}\left(n\right), \end{array}$$

where $B_{\mathrm{bd}}≜\mathrm{blkdiag}\left\{B_{\mathrm{bd},1},\ldots ,B_{\mathrm{bd},K}\right\}$ and $L_{\mathrm{bd}}\left(n\right)≜\mathrm{blkdiag}\left\{L_{\mathrm{bd},1}\left(n\right),\ldots ,L_{\mathrm{bd},K}\left(n\right)\right\}$ denote the relay transmitter and the receiver with $B_{\mathrm{bd},k}\in C^{\frac{N_{r}}{K}\times N_{s}}$ and $L_{\mathrm{bd},k}\left(n\right)≜{\left({\bar{H}}^{H}\left(n\right)\bar{H}\left(n\right)+\sigma_{n}^{2}I_{N_{s}}\right)}^{-1}{\bar{H}}_{k}^{H}\left(n\right)\in C^{N_{s}\times \frac{N_{r}}{K}}$ being their *k*-th diagonal sub-blocks, respectively. Based on the aforementioned definitions, we can, respectively, rewrite the received signals at Bob and Eve as

(11) $$\begin{array}{cc} y_{B}\left(n\right) & =\sum_{k=1}^{K}G_{k}B_{\mathrm{bd},k}L_{\mathrm{bd},k}\left(n\right)y_{R_{k}}\left(n\right)+n_{B}\left(n\right) \end{array}$$

(12) $$\begin{array}{cc} y_{E,2}\left(n\right) & =\sum_{k=1}^{K}T_{k}B_{\mathrm{bd},k}L_{\mathrm{bd},k}\left(n\right)y_{R_{k}}\left(n\right)+n_{E}\left(n\right). \end{array}$$

Now, we design the relay precoder such that $B_{\mathrm{bd},k}=G_{k}^{†}K$ for an arbitrary matrix $K\in C^{N_{b}\times N_{s}}$ to satisfy the following equations

(13) $$\begin{array}{c} G_{1}B_{\mathrm{bd},1}=\ldots =G_{K}B_{\mathrm{bd},K}=K, \end{array}$$

where $G_{k}^{†}≜G_{k}^{H}{\left(G_{k}G_{k}^{H}\right)}^{-1}$ denotes the pseudo inverse of $G_{k}$. Then, one can easily verify that Bob’s received signal in (11) is alternatively expressed as a multiplication of the precoded second hop channel K, the instantaneous Wiener filter receiver $\bar{L}\left(n\right)={\left({\bar{H}}^{H}\left(n\right)\bar{H}\left(n\right)+\sigma_{n}^{2}R_{s}^{-1}\right)}^{-1}{\bar{H}}^{H}\left(n\right)$ that equalizes the first hop effective channel $\bar{H}\left(n\right)$, and the relay received signal $y_{R}\left(n\right)$ as

(14) $$\begin{array}{c} y_{B}\left(n\right)=K\sum_{k=1}^{K}L_{\mathrm{bd},k}\left(n\right)y_{R_{k}}\left(n\right)+n_{B}\left(n\right)=K\bar{y}\left(n\right)+n_{B}\left(n\right), \end{array}$$

where $\bar{y}\left(n\right)=\bar{L}\left(n\right)y_{R}\left(n\right)$.

Now, we observe that the proposed design in (10) with a condition in (13) enables the propagation of random AFF in $\bar{H}\left(n\right)$ towards Bob’s channel is effectively blocked by the Wiener filter receiver L(n). In contrast, it is still conveyed to Eve’s channel as the fast faded channel $\bar{H}\left(n\right)$ remains unequalized in Eve’s signal $y_{E,2}$ in () as we have $T_{i}B_{\mathrm{bd},i}\neq T_{j}B_{\mathrm{bd},j},\forall i\neq j$ with probability 1.

To be more specific, let us define ${\bar{R}}_{y}≜E\left[\bar{y}{\bar{y}}^{H}|\Omega \right]$. Then, based on the result in (14), and following a similar approach to that in Lemma 1, we can formulate the MMSE problem for given the relay transceiver in (10) as

(15) $$\begin{array}{cc} \min_{P,Q,W} & {E[∥z-s∥}^{2}]=\min_{P,Q,W}E_{\Omega }\left[E[∥z-s{∥}^{2}|\Omega \right]\\ \; & =\min_{P,K,W}E_{\Omega }[E\left[∥\bar{y}-s{∥}^{2}|\Omega \right]+E\left[∥z-\bar{y}{∥}^{2}|\Omega \right]]\\ \; & =\min_{P,K,W}E_{\Omega }[E\left[∥\bar{L}\left(\bar{H}s+n_{R}\right)-s{∥}^{2}|\Omega \right]+E\left[∥W\left(K\bar{y}+n_{B}\right)-\bar{y}{∥}^{2}|\Omega \right]]\\ \; & \overset{\left(a\right)}{=}\min_{P,K}\left\{E_{\Omega }\left[\mathrm{Tr}\left({\left(\sigma_{n}^{-2}{\bar{H}}^{H}\bar{H}+R_{s}^{-1}\right)}^{-1}\right)\right]+E_{\Omega }\left[\mathrm{Tr}\left({\left(\sigma_{n}^{-2}K^{H}K+{\bar{R}}_{y}^{-1}\right)}^{-1}\right)\right]\right\}, \end{array}$$

where the equality (a) is obtained by applying the optimal destination receiver for a given random precoder Ω(n) as

(16) $$\begin{array}{c} W\left(n\right)={\left(K^{H}K+\sigma_{n}^{2}{\bar{R}}_{y}^{-1}\left(n\right)\right)}^{-1}K^{H}. \end{array}$$

The optimal receiver in (16) is prohibited in our system as it requires Bob to have knowledge on the random precoder sequence Ω(n) as well as the first hop channel HP. Nevertheless, we can devise a suboptimal receiver by invoking the high SNR approximation ${\bar{R}}_{y}\left(n\right)≃R_{s}$ as

(17) $$\begin{array}{c} W≃{\left(K^{H}K+\sigma_{n}^{2}R_{s}^{-1}\right)}^{-1}K^{H}, \end{array}$$

which remains constant over a coherence block length. Note that the approximation ${\bar{R}}_{y}\left(n\right)≃R_{s}$ holds in each symbol index *n* as we have

$$\begin{array}{cc} & \lim_{\sigma_{n}^{2}\to 0}{\bar{R}}_{y}\left(n\right)\\ \; & =\lim_{\sigma_{n}^{2}\to 0}\bar{L}\left(n\right)\left(\bar{H}\left(n\right)R_{s}{\bar{H}}^{H}\left(n\right)+\sigma_{n}^{2}I_{N_{r}}\right){\bar{L}}^{H}\left(n\right)\\ \; & =\lim_{\sigma_{n}^{2}\to 0}R_{s}{\bar{H}}^{H}\left(n\right){\left(\bar{H}\left(n\right)R_{s}{\bar{H}}^{H}\left(n\right)+\sigma_{n}^{2}I_{N_{r}}\right)}^{-1}\bar{H}\left(n\right)R_{s}\\ \; & =\lim_{\sigma_{n}^{2}\to 0}{\left({\bar{H}}^{H}\left(n\right)\bar{H}\left(n\right)+\sigma_{n}^{2}R_{s}^{-1}\right)}^{-1}{\bar{H}}^{H}\left(n\right)\bar{H}\left(n\right)R_{s}\\ \; & =R_{s}, \end{array}$$

where the second and third equalities follow from the well-known Woodbury matrix identity [40].

It is seen from (17) that with the proposed design, Bob is still able to estimate the input signal s without being affected by the first hop AFF channel $\bar{H}$. However, it is noteworthy that this is not the case for Eve. In the same manner, we can show that the joint MMSE problem in (15) can be reformulated as

(18) $$\begin{array}{cc} \min_{P,Q,W} & E[∥z-s{∥}^{2}]\\ \; & ≃\min_{P}E_{\Omega }\left[\mathrm{Tr}\left({\left(\sigma_{n}^{-2}{\bar{H}}^{H}\bar{H}+R_{s}^{-1}\right)}^{-1}\right)\right]+\min_{K}\mathrm{Tr}\left({\left(\sigma_{n}^{-2}K^{H}K+R_{s}^{-1}\right)}^{-1}\right), \end{array}$$

which is a sum of two independent problems with respect to the source and relay precoders P and K, respectively.

For security reasons, the source precoder P should be designed so as not to compromise the randomness of the AFF precoder Ω, which implies that P remains constant over time while possibly being customized to H. There is currently no known analytical solution for this problem. However, with this condition and considering the source power constraint $E[∥x_{A}{∥}^{2}]\leq P_{A}$, we can invoke the eigen-beamforming method $P=\sqrt{\frac{P_{A}}{N_{s}}}V_{h}$ as an effective source precoder, where $V_{h}\in C^{N_{a}\times N_{s}}$ denotes the right singular matrix corresponding to the $N_{s}$ largest singular values of H. Such a precoder can be particularly useful when $N_{a}>N_{s}$ as it prevents power from being wasted on the channel paths having the smallest gains.

In the meantime, as for the design of K, we can formulate an MMSE problem as

$$\begin{array}{cc} \left(P\text{-}1\right)\;\min_{K} & \mathrm{Tr}({\left(\sigma_{n}^{-2}K^{H}K+R_{s}^{-1}\right)}^{-1})\\ s.t. & \sum_{k=1}^{K}Tr\left(G_{k}^{†}KR_{y_{k}}K^{H}G_{k}^{†H}\right)\leq P_{R}, \end{array}$$

where $R_{y_{k}}≜E\left[y_{k}y_{k}^{H}\right]$ with $y_{k}≜L_{\mathrm{bd},k}y_{R_{k}}$. Note that the constraint of (P-1) follows from the maximum power limit at the relay, i.e., $E[∥Qy_{R}{∥}^{2}]\leq P_{R}$.

The problem (P-1) is generally non-convex, and thus it is difficult to find the globally optimal solution. In the subsequent subsections, we propose two distinct approaches to address this problem. The first is a GD-based iterative method that yields a locally optimal solution, while the second is a convex relaxation method which provides an insightful closed-form solution. Note that the GD-based solution can be close to the global optimum with multiple initial points, whereas the closed-form solution exhibits sub-optimal performance with significantly reduced computational complexity.

Without loss of optimality, we can set $K=\beta \bar{K}$, where β denotes a power-normalizing coefficient, which is given by

(19) $$\begin{array}{c} \beta =\sqrt{\frac{P_{R}}{\sum_{k=1}^{K}\mathrm{Tr}\left(G_{k}^{†}\bar{K}R_{y_{k}}{\bar{K}}^{H}G_{k}^{†H}\right)}}. \end{array}$$

Then, the problem (P-1) can be reformulated as an unconstrained optimization problem as

$$\begin{array}{c} \left(P\text{-}2\right)\;\;\min_{\bar{K}}\Xi \end{array}$$

where Ξ is defined by $\Xi ≜\mathrm{Tr}\left(R_{K}^{-1}\right)$ with $R_{K}=\sigma_{n}^{-2}\beta^{2}{\bar{K}}^{H}\bar{K}+R_{s}^{-1}$.

### 4.2. GD-Based Optimal Design

Now, we apply a GD algorithm to solve the unconstrained optimization problem (P-2). Using some rules for the differential dTr(Y)=Tr(dY) and $dY^{-1}=-Y^{-1}dYY^{-1}$, we can compute the differential of the MSE Ξ with respect to ${\bar{K}}^{*}$ as

$$\begin{array}{cc} d\Xi & =-\mathrm{Tr}\left(R_{K}^{-1}dR_{k}R_{K}^{-1}\right)\\ \; & =-\mathrm{Tr}\left(\frac{\beta^{2}}{\sigma_{n}^{2}}R_{k}^{-2}d{\bar{K}}^{H}\bar{K}+\frac{d\beta^{2}}{\sigma_{n}^{2}}R_{K}^{-2}{\bar{K}}^{H}\bar{K}\right)\\ \; & =-\mathrm{Tr}\left(\frac{\beta^{2}}{\sigma_{n}^{2}}\bar{K}R_{k}^{-2}d{\bar{K}}^{H}\right)+\frac{\beta^{4}}{P_{R}\sigma_{n}^{2}}\mathrm{Tr}\left(R_{K}^{-2}\bar{K}{\bar{K}}^{H}\right)\mathrm{Tr}\left(\sum_{k=1}^{K}{\left(G_{k}G_{k}^{H}\right)}^{-1}\bar{K}R_{y_{k}}d{\bar{K}}^{H}\right). \end{array}$$

For a real valued function f(·), the gradient of *f* with respect to a complex matrix Z is defined by a partial derivative $\nabla_{Z}f≜2\frac{\partial f}{\partial Z^{*}}$. Also, when the differential of *f* is given by $df=\mathrm{Tr}(A_{0}dZ+A_{1}dZ^{H})$, the gradient can be computed as $\frac{\partial f}{\partial Z^{*}}=A_{1}$ [41]. By invoking these rules, the gradient of the MSE Ξ with respect to $\bar{K}$ can be derived as

(20) $$\begin{array}{cc} \nabla_{\bar{K}}\Xi & =-\frac{2\beta^{2}}{\sigma_{n}^{2}}\bar{K}R_{k}^{-2}+\frac{2\beta^{4}}{P_{R}\sigma_{n}^{2}}\mathrm{Tr}\left(R_{K}^{-2}\bar{K}{\bar{K}}^{H}\right)\sum_{k=1}^{K}{\left(G_{k}G_{k}^{H}\right)}^{-1}\bar{K}R_{y_{k}}. \end{array}$$

Now, we can solve the problem (P-2) using the proposed gradient descent algorithm, which is summarized in Algorithm 1. In this algorithm, $N_{g}$ initial points are employed to prevent the optimization process from being stuck in undesirable local minima, and ϵ denotes the tolerance factor for terminating the iteration. We determine the step size δ by using the Armijo’s rule, which is shown to provide probable convergence [42].
**Algorithm 1** Proposed GD algorithm**for** $n=1:N_{g}$ **do**   Initialize $\bar{K}={\bar{K}}^{\left(n\right)}$ for a set of $N_{g}$ initial points $\left\{{\bar{K}}^{\left(1\right)},\ldots ,{\bar{K}}^{\left(N_{g}\right)}\right\}$   **repeat**     Compute the gradient $\nabla_{\bar{K}}\Xi$ from (20).     Determine a step size δ.     Update $\bar{K}\leftarrow \bar{K}-\delta \nabla_{\bar{K}}\Xi$.   **until** $∥\nabla_{\bar{K}}{\Xi ∥}_{F}\leq \epsilon$**end for**Select the best one among the $N_{G}$ solutions.

### 4.3. Closed-Form Design

Although the aforementioned GD method provides a near optimal solution, the iterative approach may incur excessive computational complexity at the relay. In this subsection, we propose a simple closed-form solution by applying the convex-hull relaxation technique to the feasible domain. The resulting approach achieves significantly lower complexity, comparable to that of the AN-based jamming schemes and thus is practically more meaningful than GD.

To this end, let us first examine the following lemma.

**Lemma 2.**  *The consumed power at the relay in (P-1) is bounded below as*

(21) $$\begin{array}{c} \sum_{k=1}^{K}Tr\left(G_{k}^{†}KR_{y_{k}}K^{H}G_{k}^{†H}\right)\geq \alpha Tr\left(K^{H}AK\right), \end{array}$$

*where we have $A≜\sum_{k=1}^{K}{\left(G_{k}G_{k}^{H}\right)}^{-1}\in C^{N_{b}\times N_{b}}$ and $\alpha ≜\min_{k}\lambda_{\min}\left(R_{y_{k}}\right)$ with $\lambda_{\min}\left(R_{y_{k}}\right)$ denoting the minimum eigenvalue of $R_{y_{k}}$, and the resulting lower-bound in (21) is a convex function of K.*

**Proof.**  Since $R_{y_{k}}$ is a positive-definite matrix, we can establish Loewner’s order as $R_{y_{k}}⪰\lambda_{\min}\left(R_{y_{k}}\right)I_{N_{s}}$. Then, it simply follows that

$$\begin{array}{cc} \sum_{k=1}^{K}\mathrm{Tr}\left(G_{k}^{†}KR_{y_{k}}K^{H}G_{k}^{†H}\right) & \geq \sum_{k=1}^{K}\lambda_{\min}\left(R_{y_{k}}\right)\mathrm{Tr}\left(K^{H}G_{k}^{†H}G_{k}^{†}K\right)\\ \; & \geq \alpha \mathrm{Tr}\left(K^{H}AK\right). \end{array}$$

The resulting lower-bound is a convex function of K since its Hessian is given by $\nabla_{K}^{2}\mathrm{Tr}\left(K^{H}AK\right)=I_{N_{b}}⊗A$, which is positive-definite, and thus the proof is concluded. □

The result in Lemma 2 indicates that $R_{y_{k}}$ in (P-1) can be replaced with $\alpha I_{N_{s}}$, thereby relaxing the non-convex feasible domain to its nearest convex hull and rendering the problem tractable. However, the resulting solution does not necessarily satisfy the relay transmit power constraint $P_{R}$ since such a direct substitution enlarges the feasible region of K compared to the original problem. Therefore, an additional power normalization factor must be applied to the derived solution to ensure compliance with the power constraint. To address this issue, we set $K=\beta \bar{K}$ as in the previous subsection, where β serves as a power-normalizing factor as illustrated in (19). Based on the above arguments, the problem (P-1) is can then be reformulated as

$$\begin{array}{cc} \left(P\text{-}3\right)\;\;\min_{\bar{K}}\; & \mathrm{Tr}[{\left(\sigma_{n}^{-2}{\bar{K}}^{H}\bar{K}+R_{s}^{-1}\right)}^{-1}]\\ s.t.\; & \mathrm{Tr}({\bar{K}}^{H}A\bar{K})\leq P_{R}/\alpha . \end{array}$$

The above problem is now easy to solve as it is reducible to a simple scalar convex optimization problem. To see this, let us define the eigenvalue decomposition (ED) $A=U_{a}\Lambda_{a}U_{a}^{H}$, where $U_{a}\in C^{N_{b}\times N_{b}}$ and $\Lambda_{a}\in R^{N_{b}\times N_{b}}$ represent a unitary eigenvector matrix of A and a diagonal matrix with $N_{b}$ non-zero eigenvalues $\left\{\lambda_{a,1},\ldots ,\lambda_{a,N_{b}}\right\}$ on its main diagonal with a descending order, respectively. Similarly, we define $R_{s}=U_{s}\Lambda_{s}U_{s}^{H}$, where $U_{s}\in C^{N_{s}\times N_{s}}$ and $\Lambda_{s}\in R^{N_{s}\times N_{s}}$ represent the eigenvector and eigenvalue matrices with $N_{s}$ non-zero eigenvalues $\left\{\lambda_{a,1},\ldots ,\lambda_{a,N_{s}}\right\}$ with a descending order, respectively.

Then, one can easily verify that the optimal solution of (P-3) diagonalizes both the objective and constraint functions simultaneously [33], which gives rise to a solution

$$\begin{array}{c} \bar{K}={\bar{U}}_{a}\Phi U_{s}^{H}, \end{array}$$

where ${\bar{U}}_{a}\in C^{N_{b}\times N_{s}}$ and $\Phi \in C^{N_{s}\times N_{s}}$ denote a unitary beamforming matrix composed of the first $N_{s}$ columns of $U_{a}$ and a diagonal power allocation matrix with its *i*-th element being denoted by $\phi_{i}\geq 0$, respectively. Thus, the remainder is a scalar optimization problem of $\left\{\phi_{i},\forall i\right\}$, which is easily solved through the Karush–Kuhn–Tucker (KKT) conditions as

(22) $$\begin{array}{c} \phi_{i}={\left({\left(\mu \sigma_{n}\lambda_{a,i}^{-1/2}-\sigma_{n}^{2}\lambda_{s,i}^{-1}\right)}^{+}\right)}^{1/2}\;\mathrm{for}\;i=1,\ldots ,N_{s}. \end{array}$$

Here, μ≥0 represents the Lagrange multiplier that can be chosen to satisfy the constraint in problem (P-3) with equality. Finally, for given $\bar{K}$, we apply β in order to adjust the relay transmit power to its maximum value $P_{R}$. Finally, we attain a complete solution for the relay transceiver in (10) as

(23) $$\begin{array}{cc} Q_{\mathrm{bd},k}\left(n\right) & =\beta G_{k}^{†}{\bar{U}}_{a}\Phi U_{s}^{H}{\left({\bar{H}}^{H}\left(n\right)\bar{H}\left(n\right)+\sigma_{n}^{2}R_{s}^{-1}\right)}^{-1}{\bar{H}}_{k}{\left(n\right)}^{H},\;\forall k. \end{array}$$

### 4.4. Detection Process at Bob

First of all, Bob can readily estimate the desired effective channel $K=\beta {\bar{U}}_{a}\Phi U_{s}^{H}$ by exploring the information of the second-hop channel G. Then, Bob applies the approximated receiver in (17) to the received signal $y_{B}\left(n\right)$ and detects the messages through a simple symbol-by-symbol distance-based metric

$$\begin{array}{c} {\hat{s}}_{i}=\arg\min_{s_{i}\in M}{\left|z_{i}-s_{i}\right|}^{2},\;\mathrm{for}\;i=1,\ldots ,N_{s} \end{array}$$

without being affected by the random fluctuation of the first-hop effective channel $\bar{H}\left(n\right)$. Here, $z_{i}$ and $s_{i}$ denote the *i*-th element of z and s, respectively, and M represents a set of modulation symbols.

### 4.5. Detection Process at Eve

By combining the received signals over the two time slots, Eve effectively obtains

(24) $$\begin{array}{c} y_{E}\left(n\right)=T_{\mathrm{aff}}\left(n\right)s\left(n\right)+n_{\mathrm{aff}}\left(n\right), \end{array}$$

where we have

$$\begin{array}{c} T_{\mathrm{aff}}\left(n\right)=\left[\begin{array}{c} FP\\ TQ(n)HP \end{array}\right]\Omega \left(n\right)\;\;\mathrm{and}\;\;n_{\mathrm{aff}}\left(n\right)=\left[\begin{array}{c} n_{E,1}\left(n\right)\\ TQ\left(n\right)n_{R}\left(n\right)+n_{E,2}\left(n\right) \end{array}\right]. \end{array}$$

Also, as in the case of AN, we assume that Eve can accurately estimate its ENC. Then, we apply the MCA technique to $y_{E}\left(n\right)$ in order to mitigate the effect of possible noise and interference terms as

(25) $$\begin{array}{cc} y_{E}^{\prime }\left(n\right) & =W_{\mathrm{mca}}y_{E}\left(n\right)={\bar{T}}_{\mathrm{aff}}\left(n\right)s\left(n\right)+n_{w}\left(n\right), \end{array}$$

where ${\bar{T}}_{\mathrm{aff}}\left(n\right)=W_{\mathrm{mca}}T_{\mathrm{aff}}\left(n\right)$ with $W_{\mathrm{mca}}$ being obtained from the estimated ENC as in (8).

It is easily inferred from (25) that the proposed AFF design is inherently robust to ANE attacks because the jamming noise is multiplied to the information bearing signals rather than being added as interference. It is also noteworthy that even if the CSI of the wiretap channels F and T is known to Eve, Eve is still impaired by the absence of knowledge regarding the random precoder sequence ${\left\{\Omega \left(n\right)\right\}}_{n=1}^{N}$ as well as the legitimate link CSI H and G. In this situation, the best strategy available at Eve is to adopt the blind estimation schemes. Unlike the case of AN, however, the random precoder Ω varying over short intervals will continuously hinder Eve’s blind estimation even after the MCA is applied. In particular, if Ω changes in every symbol time, i.e., T=1, Eve’s error rate can be non-negligibly high, although it may increase the short-term computational complexity at the relay.

In terms of maximum likelihood (ML), the blind estimation is performed as [43,44]

(26) $$\begin{array}{cc} \hat{S} & =\arg\min_{S\in M^{NN_{s}}}\min_{{\bar{T}}_{\mathrm{aff}}}{∥Y_{E}^{\prime }-{\bar{T}}_{\mathrm{aff}}S∥}_{F}^{2}\\ \; & =\underset{S\in M^{NN_{s}}}{arg max}\mathrm{Tr}\left\{Y_{E}^{\prime }S^{H}{\left(SS^{H}\right)}^{-1}SY_{E}^{\prime H}\right\}, \end{array}$$

which can be found by an exhaustive search over the entire symbol space ${\left|M\right|}^{NN_{s}}$ with |M| denoting the number of symbols in M. This incurs high search complexity, but the use of sphere decoding may alleviate the computational burden to some extent [43]. Such a blind technique works well in the case where Eve’s effective channel ${\bar{T}}_{\mathrm{aff}}$ remains constant over a relatively long period of time, e.g., T≥N. In contrast, as *T* decreases, the accuracy of signal detection gradually degrades. Our statement so far will be demonstrated in Section 5 via simulation results.

### 4.6. Complexity Analysis

In this subsection, we briefly analyze the required computational complexity of the non-secure, AN-based secure, and the proposed AFF-based secure MMSE relaying designs at each node. Note that for fair comparison and practical relevance, the computational complexity of all three techniques is analyzed based on their respective closed-form solutions. In general, a single real-valued addition or multiplication is counted as one floating point operation (flop). As a result, a complex addition and a complex multiplication require 2 and 6 flops, respectively, which implies that the number of multiplications in the complex domain dominates overall complexity.

For simplicity, in this paper, we count each complex multiplication as one flop with any additive and real valued operations being omitted. For operations such as ED and matrix inversion (MI), it is also cumbersome to precisely quantify the exact computational cost. Thus, the associated complexity is characterized in terms of the order of growth $O_{\mathrm{ED}}\left(\cdot \right)$ and $O_{\mathrm{MI}}\left(\cdot \right)$ with respect to the matrix dimension, respectively. In addition, some transmit and receive filters need to be updated only once over the channel coherence time. Therefore, the computational complexity can be evaluated from two perspectives, namely, symbol-level short-term and coherence-time-level long-term complexity.

The results of our complexity analysis are summarized in Table 2. It can be observed that compared with the conventional non-secure and AN-based secure designs, the proposed AFF exhibits a noticeable complexity increase at the relay, particularly from the short-term perspective. A major part of the complexity increase stems from the symbol-level computation of the relay receiver $L_{\mathrm{bd}}\left(n\right)$, including a matrix computation ${\bar{H}}^{H}\left(n\right)\bar{H}\left(n\right)$ and a $N_{s}\times N_{s}$ matrix inversion. However, performing these computations within a symbol duration, which is usually several tens of μs [45], could be challenging. It is also worth noting that the proposed relay-side receiver assumes perfect synchronization of the random precoders between Alice and the relay.

One notable feature of the proposed AFF scheme is that the source and the relay can locally generate and pre-share a set of the random precoder sequence in advance. In this case, the relay does not need to compute filters at every symbol interval. Instead, all transmit and receive filters to be used over the coherence time can be pre-computed at the beginning of each coherence block. Then, at each symbol time, the relay simply selects and applies the corresponding pre-computed filter. In this manner, the proposed approach can further reduce the short-term complexity to the level of non-secure designs. Such a characteristic is distinguished from existing symbol-level precoding approaches [36,46], which requires symbol-level precoder computation depending on the modulated symbols. Meanwhile a rigorous robustness analysis under imperfect or delayed precoding conditions would require explicit modeling of precoder mismatch and synchronization errors. Thus, the development of robust receiver designs that can tolerate such impairments remains an open and meaningful extension of the proposed framework.

## 5. Simulation Results

In this section, we present numerical simulation results to demonstrate the effectiveness of the proposed AFF designs. For ease of exposition, we set $P_{A}=P_{R}=P_{0}$ and define $\mathrm{SNR}=P_{0}/\sigma_{n}^{2}$ throughout the section. We adopted a Rayleigh block fading channel model, where all channel coefficients are generated according to a complex Gaussian distribution CN(0,1) for each coherence block, which implies that all inter-node distances are considered to be identical. Further, Eve observes the same signals redundantly over two time slots unlike Bob, which effectively doubles the number of antennas. Thus, the model configures a channel environment that is favorable to Eve rather than Bob.

We consider uncoded transmission from Alice with $R_{s}=I_{N_{s}}$ in order to isolate the impact of the proposed design on the detection performance. Also, we use the binary phase shift keying (BPSK) modulation scheme with K=2, N=25, and T=1 unless stated otherwise. With regard to the AFF precoder, we choose each element of Ω from the i.i.d. Gaussian distribution such that $\Omega ∼\mathrm{CN}(0,\sigma_{\omega }^{2})$, which was shown to minimize the leakage information to Eve [23]. Here, we set $\sigma_{\omega }^{2}=1/\sqrt{N_{s}}$ to meet the design condition $E\left[\Omega \Omega^{H}\right]=I_{N_{s}}$.

In our simulation, it is assumed that Eve attains prior knowledge on the first *U* symbol vectors, i.e., U=[s(1),…,s(U)] out of *N* symbols in S in each coherence block. Such an assumption captures a realistic threat model where Eve leverages prior knowledge of the underlying communication protocol or standard. For example, when Eve is aware of the signal field or control channel structures in a data packet, it may be able to infer certain messages such as control information contained within it. Therefore it is important to observe Eve’s detection behavior according to the amount of messages leaked to Eve.

With this prior knowledge, Eve first estimates its ENC by invoking the ML-based ENC estimation process. Further, we consider that Eve is equipped with a sufficiently large number of antennas such that $N_{e}\geq \left\lceil \frac{1}{2}\left(M_{E}+N_{s}\right)\right\rceil$ to perform ANE. Note that it can be numerically verified that in terms of Eve, $M_{E}=M$ and $2N_{s}$ exhibit the best performance for AN and AFF schemes, respectively. Based on these capabilities, Eve applies MCA to the received signals as in (9) and (25) to attenuate the effect of noise. Finally, it attempts to intercept the messages from *N* observed signals $Y_{E}^{\prime }=\left[y_{E}^{\prime }\left(1\right),\ldots ,y_{E}^{\prime }\left(N\right)\right]$ through the blind estimation method in (26). All BER results in this section were obtained by averaging over $10^{6}$ Monte Carlo trials and employ a fixed error-count stopping criterion of 1000 observed errors, resulting in consistent confidence interval widths across different scenarios.

**Figure 2:**
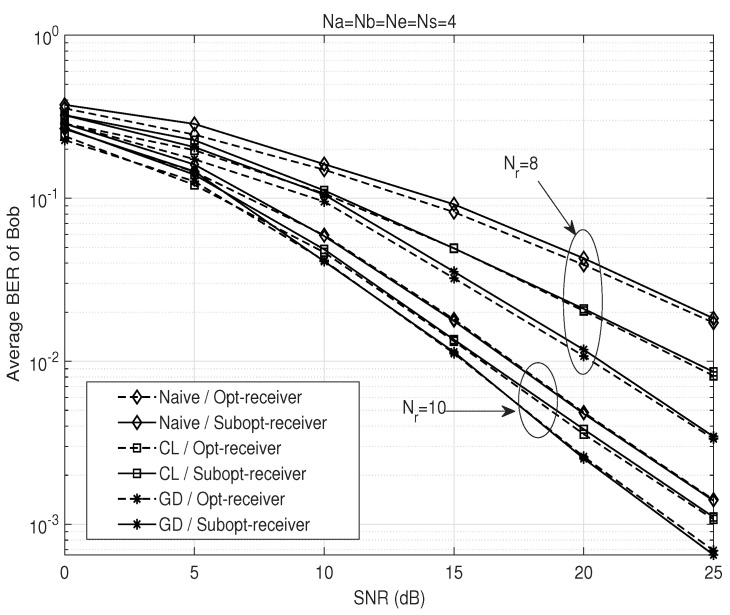
BER performance comparison of the proposed AFF schemes at Bob with respect to SNR (dashed lines: suboptimal receiver, solid lines: optimal receiver).

Figure 2 depicts the BER performance of the proposed AFF scheme at Bob in $N_{a}=N_{b}=N_{e}=N_{s}=4$ and $N_{r}=8$ wiretap relay channels. From this figure, we confirm that the proposed optimization schemes such as the GD and closed-form (CL) methods achieve substantial performance gains compared to a naive solution $K=\beta I_{N_{b}\times N_{s}}$. While the performance gain gradually diminishes as $N_{r}$ increases, a diversity gain is observed for all schemes. It is also demonstrated that over the entire SNR range, the suboptimal receiver in (17) achieves performance close to the optimal scheme in (16) despite being independent of the first-hop channel. For this reason, in the following, we consider the CL with a suboptimal receiver as a representative AFF scheme, which is more practical than the optimal designs and achieves a performance gain over the naive scheme.

**Figure 3:**
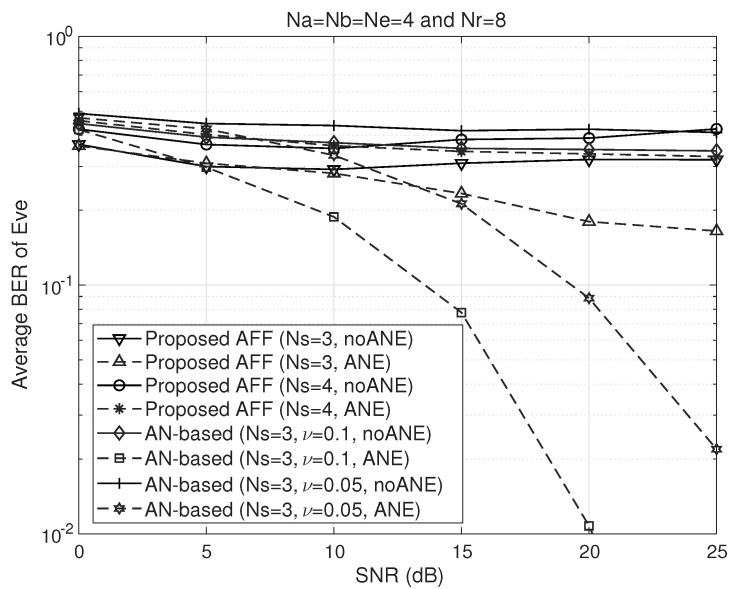
BER performance comparison of the proposed AFF and AN schemes at Eve with respect to SNR (dashed lines: with ANE, solid lines: without ANE).

Figure 3 illustrates the BER performance of the AN and AFF schemes at Eve in $N_{a}=N_{b}=N_{e}=4$ and $N_{r}=8$ wiretap relay channels. In this figure, we assumed that Eve has U=20 prior knowledge, which enables Eve to estimate its ENC with sufficient accuracy to perform ANE. In addition, we consider $N_{s}=3$ and 4 spatial multiplexing transmission scenarios with and without ANE at Eve. As for the AN-based design, we set ν=0.1 and 0.05, which implies that 90% and 95% of the transmit power is allocated to the artificial noise, respectively. The simulation results demonstrate that while the proposed AFF is robust to ANE attack, the AN-based jamming scheme is highly vulnerable to such an attack even in the case of high AN power. In contrast, as the signal power ratio, i.e., ν, increases, this vulnerability deteriorates more rapidly. From the BER plots obtained without ANE, one can observe slight fluctuations with increasing SNR. This behavior is explained by the absence of ANE, whereby higher SNR levels also amplify the effect of interference.

**Figure 4:**
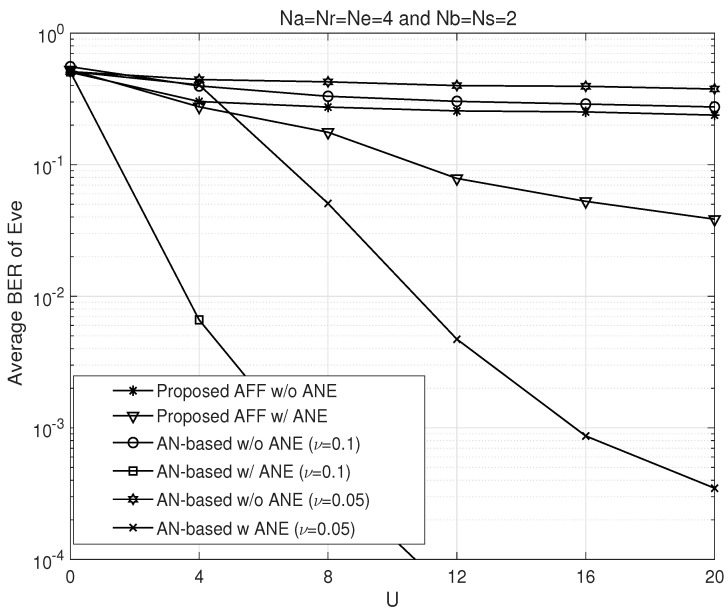
BER performance comparison of the proposed AFF and AN schemes at Eve with respect to Eve’s prior knowledge *U*.

In Figure 4, we examine the effect of Eve’s prior knowledge *U* on the detection probability in $N_{a}=N_{r}=N_{e}=4$ and $N_{b}=N_{s}=2$ wiretap channels. As observed in the figure, when the amount of leaked a priori information *U* is relatively small, both the AN and AFF schemes appear to be secure. However, as *U* increases, the probability of information leakage gradually increases. This behavior is attributed to the improved accuracy of ENC estimation with increasing *U*. One notable point is that as both *U* and ν increase, the detection probability of the AN scheme rises more rapidly than that of the AFF scheme.

**Figure 5:**
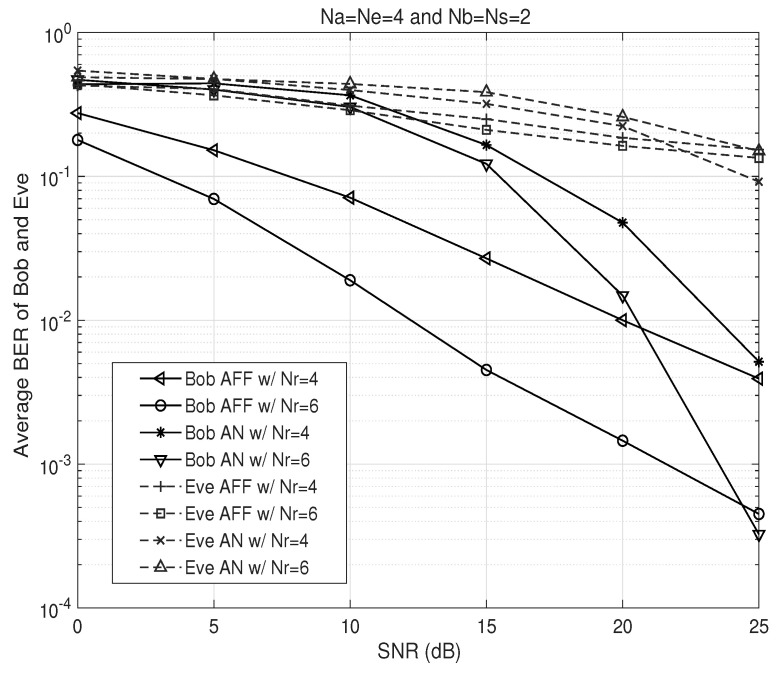
BER performance comparison of the proposed AFF and AN schemes at both Bob and Eve with respect to SNR (dashed lines: Eve, solid lines: Bob).

Figure 5 illustrates the BER performance of Bob and Eve in $N_{a}=N_{e}=4$ and $N_{b}=N_{s}=2$ wiretap relay channels with various $N_{r}$. We see that U=10 and ν=0.02 for the AN-based design. This figure shows that, for the AN-based design, up to 98% of the maximum transmit power must be allocated to AN in order to achieve a security level comparable to that of the proposed AFF scheme. Moreover, it can be observed that, at the same security level, the proposed AFF scheme provides improved detection performance for Bob compared to the AN scheme.

**Figure 6:**
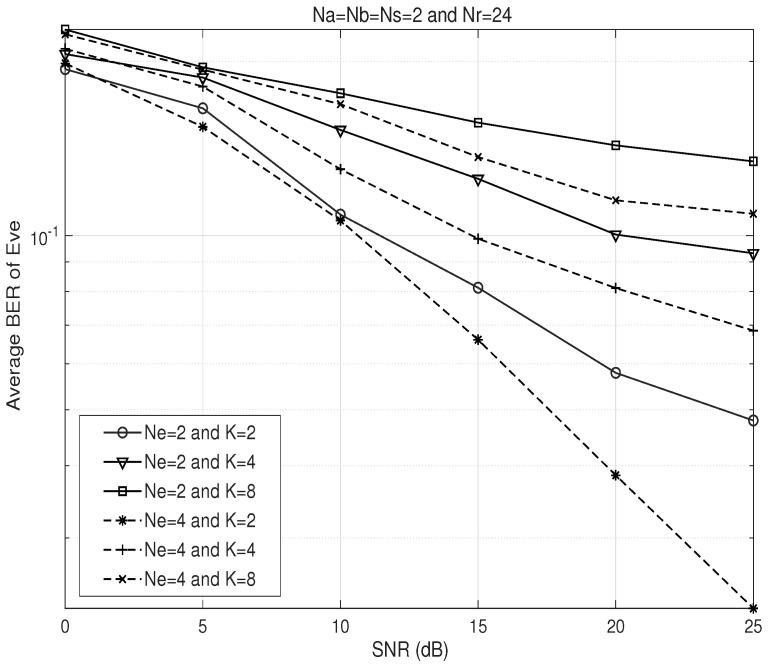
BER performance comparison of the proposed AFF scheme at Eve with respect to SNR (dashed lines: $N_{e}=4$, solid lines: $N_{e}=2$).

Figure 6 compares the BER performance of the proposed AFF scheme at Eve in $N_{a}=N_{b}=N_{s}=2$ and $N_{r}=24$ wiretap relay channels. Here, we examine how the security performance of the proposed AFF scheme varies with the parameter *K*. It is confirmed from the figure that as *K* increases, the security level enhances regardless of the number of Eve’s antennas $N_{e}$. However, it is noteworthy that security comes at the cost of diversity loss of Bob due to reduced degrees of freedom at the relay.

**Figure 7:**
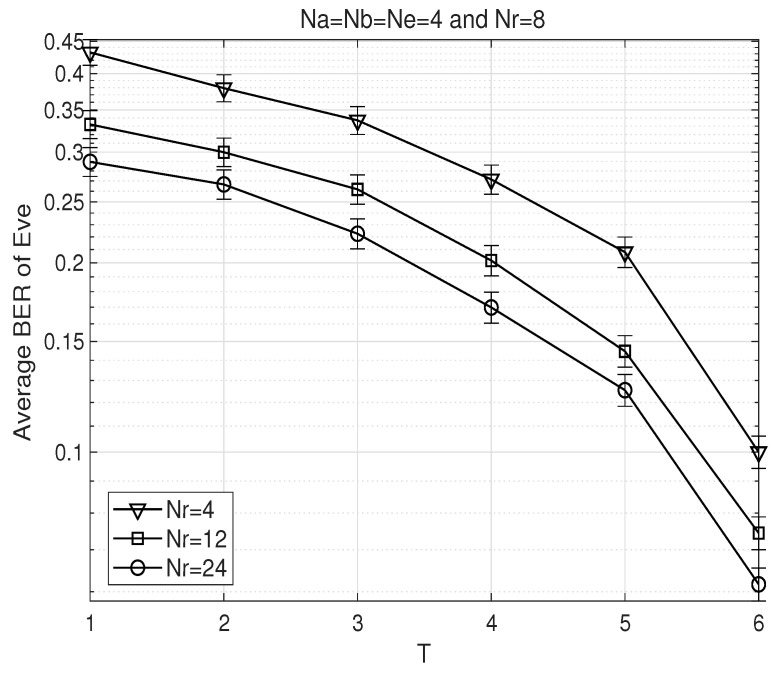
BER performance comparison of the proposed AFF scheme at Eve with respect to the effective coherence time *T*.

Figure 7 exhibits the BER performance of the proposed AFF scheme at Eve with respect to the effective channel coherence time *T*. In this figure, we set U=1 considering a typical Eve that captures little priory information from the received data frame. In this case, the accuracy of ENC estimation is significantly degraded, and thus ANE hardly operates effectively. To exclude the malfunction of ANE, this figure examines the variation in Eve’s error rate with respect to the rate of the AFF precoder in the absence of ANE. It can be observed that the system remains highly secure when *T* is small, whereas the security threat to the proposed AFF scheme increases as *T* grows. As discussed in the complexity analysis in Section 4.6, reducing the variation interval *T* may increase the computational complexity at the relay. However, in scenarios where the AFF precoder can be generated locally in advance, all the required filters can be precomputed, thereby alleviating the implementation complexity to some extent even for small values of *T*. In this figure, the confidence intervals are illustrated as error bars to demonstrate the statistical reliability of the Monte Carlo simulation results. Specifically, 95% confidence intervals were computed based on the total number of transmitted bits and observed errors using the exact Clopper–Pearson method [47] for binomial distribution. Although not presented in the previous figures, all other BER results exhibit confidence intervals of comparable width for the same simulation settings.

## 6. Conclusions and Future Works

In this paper, we have proposed a novel AFF design for MMSE-based spatial multiplexing MIMO AF relay systems to enhance physical-layer security. The key idea is to introduce a random precoder at the transmitter that deliberately randomizes the relay channel, thereby inducing artificial fast fading at the eavesdropper while maintaining reliable communication for the legitimate user. By jointly optimizing the transmit and receive filters at each node for the MMSE criterion, the proposed scheme effectively mitigates the impact of precoder randomness on Bob’s reception while significantly deteriorating Eve’s detection performance. The proposed approach achieves LPI without requiring modification to the standard training structure of conventional MMSE-based relaying systems, making it readily applicable to existing frameworks. Simulation results confirm that the designed AFF-based scheme successfully suppresses information leakage to the eavesdropper while guaranteeing communication quality for the legitimate receiver.

Future work will focus on extending the analysis toward theoretical evaluation of the secrecy rate, providing a quantitative measure of the achievable secure throughput. One may also extend this framework to more practical scenarios such as the existence of partial correlation between the legitimate and wiretap channels, channel estimation and synchronization errors, and user mobility, thereby further strengthening its robustness against advanced eavesdropping strategies. It is also noteworthy that the proposed AFF scheme is primarily designed for relay-assisted scenarios, where the direct path between Alice and Bob does not exist due to high path loss. As we assume that Bob does not share a secret key with Alice, Bob is unable to leverage the direct signals from Alice, even if there exists a non-negligible direct link. An extension of our scheme to the scenarios of utilizing the direct link will also be an interesting topic for future works.

## Figures and Tables

**Table 1:** The amount of CSI at each node.

Alice	Relay	Bob	Eve
H	H, G	G	F, T

**Table 2:** Computational complexity comparison.

	Source		Relay	
	Short-Term	Long-Term	Short-Term	Long-Term
Unsecured	$N_{a}N_{s}$	$O_{\mathrm{ED}}\left(N_{a}^{3}\right)$	$2N_{r}N_{s}$	$\begin{array}{c} O_{\mathrm{MI}}\left(N_{s}^{3}\right)+O_{\mathrm{ED}}\left(N_{r}^{3}\right)\\ +N_{r}\left(N_{s}^{2}+N_{r}N_{s}\right) \end{array}$
AN-based	$N_{a}^{2}$		$N_{r}\left(N_{r}+Ns\right)$	$\begin{array}{c} O_{\mathrm{MI}}\left(N_{s}^{3}\right)+O_{\mathrm{ED}}\left(N_{r}^{3}\right)\\ +N_{r}\left(N_{s}^{2}+N_{r}N_{s}\right) \end{array}$
AFF-based	$N_{s}\left(N_{a}+N_{s}\right)$		$\begin{array}{c} O_{\mathrm{MI}}\left(N_{s}^{3}\right)+N_{s}\left(2N_{r}+N_{s}\right)\\ +N_{s}^{2}N_{r} \end{array}$	$\begin{array}{c} KO_{\mathrm{MI}}\left(N_{b}^{3}\right)+O_{\mathrm{ED}}\left(N_{b}^{3}\right)\\ +N_{r}\left(3N_{b}^{2}+N_{b}N_{s}+N_{r}N_{s}+N_{s}^{2}+N_{s}\right) \end{array}$

## Data Availability

Data are contained within the article.
